# Recent Advances in Anti-HCV, Anti-HBV and Anti-Flavivirus Agents

**DOI:** 10.3390/v18010020

**Published:** 2025-12-23

**Authors:** Grigoris Zoidis

**Affiliations:** Division of Pharmaceutical Chemistry, Department of Pharmacy, School of Health Sciences, National and Kapodistrian University of Athens, Panepistimiopolis-Zografou, 15771 Athens, Greece; zoidis@pharm.uoa.gr; Tel.: +30-210-7274809

Viral infections have shaped human history since the earliest stages of civilization and continue to exert one of the greatest global pressures on health, socioeconomic stability, and public health infrastructures. Even with major advances in immunology, vaccinology, and antiviral therapeutics, viruses remain responsible for millions of deaths each year and for recurrent outbreaks of international concern. This persistence reflects both the extraordinary adaptability of viral pathogens and the enduring inequities in access to prevention, diagnosis, and treatment worldwide.

Chronic viral hepatitis remains among the most consequential causes of infectious disease mortality. The 2024 Global Hepatitis Report estimated 1.3 million deaths from hepatitis B virus (HBV) and hepatitis C virus (HCV) in 2022, positioning viral hepatitis among the world’s deadliest infections [1]. This toll persists despite curative therapies for HCV and the availability of a safe, highly effective HBV vaccine. Underdiagnosis, insufficient linkage to care, and pronounced disparities in treatment access continue to drive this burden, as emphasized across multiple landmark analyses [2,3,4].

At the same time, arthropod-borne flaviviruses have re-emerged as major global threats. Dengue virus (DENV), responsible for an estimated 390 million infections each year, has expanded dramatically under the combined influence of climate change, globalization, and vector adaptation [5,6]. Zika virus (ZIKV) has caused congenital and neurological complications on an unprecedented scale [7], while West Nile virus (WNV) and Yellow Fever virus (YFV) continue to generate severe outbreaks, with case fatality rates reaching 20–30% in some settings [8,9]. Despite this escalating burden, no specific antiviral therapy has been approved for DENV, ZIKV, WNV, or YFV—an increasingly urgent gap noted in several recent reviews [10]. Reflecting this urgency, the WHO has designated Zika virus as priority pathogen for accelerated research and development [11].

Compounding these challenges, the evolution of antiviral resistance remains a persistent threat. Although direct-acting antivirals (DAAs) have transformed HCV treatment, resistance-associated substitutions can undermine therapeutic efficacy, particularly in resource-limited regions lacking optimal drug combinations or diagnostic tools. Sustained surveillance for resistance and the development of next-generation agents remain essential to long-term viral hepatitis control [12,13].

In response to these converging global pressures, antiviral research has entered a period of rapid innovation. Current efforts increasingly extend beyond classical viral enzymes to include host pathways that support viral entry, replication, and assembly. Broad-spectrum compounds, host-directed therapies, monoclonal antibodies, RNA-based interventions, and structure-guided small-molecule scaffolds represent a strategic shift toward more resilient antiviral preparedness [14]. The ability to anticipate viral evolution, overcome resistance, and respond rapidly to emerging threats will be central to future public health security.

Together, these realities underscore the pressing need to accelerate the discovery and development of next-generation antiviral agents. Expanding mechanistically novel therapeutics and strengthening the antiviral pipeline are essential to confront long-standing pathogens such as HBV and HCV, as well as rapidly emerging flaviviruses including DENV, ZIKV, and WNV.

The first paper in this Special Issue is a review that provides an in-depth analysis of the structural and functional properties of the hepatitis B virus (HBV) *ε* (epsilon) signal (https://doi.org/10.3390/v15091913 URL [15]), a highly conserved RNA element indispensable for viral replication. ε is a multifunctional cis-acting motif positioned at both the 5′ and 3′ ends of the pregenomic RNA (pgRNA), where it governs the encapsidation, reverse transcription, and recruitment of the viral polymerase. Structural investigations show that ε comprises a lower stem, an apical loop, and a central bulge—the latter serving as the critical platform for HBV polymerase (P protein) engagement and the initiation of minus-strand DNA synthesis. The review highlights that the bulge and upper stem constitute the principal determinants of polymerase recognition, whereas the apical loop contributes predominantly to structural stability. Comparative analyses across HBV genotypes reveal that, despite extensive sequence variation, the secondary structure of ε remains strikingly conserved, reflecting strong evolutionary pressure. Mutagenesis studies further demonstrate that even subtle modifications in bulge geometry or stem integrity profoundly impair pgRNA packaging and polymerase priming, underscoring the finely tuned structural requirements of HBV replication. These insights reinforce ε as an attractive antiviral target, particularly for strategies aimed at disrupting RNA–protein interactions or modulating RNA folding. Overall, the work illuminates how a compact RNA motif orchestrates multiple steps of the HBV life cycle through highly specific structural features.

The second paper (https://doi.org/10.3390/v15122367 [16]) is a comprehensive review that examines the development of sulfamoyl-based capsid assembly modulators (CAMs) as emerging antiviral agents directed against hepatitis B virus (HBV). HBV persistence is driven by covalently closed circular DNA (cccDNA), which is not eliminated by existing nucleos(t)ide analog therapies. CAMs interfere with nucleocapsid assembly, thereby blocking pgRNA encapsidation, reverse transcription, and the generation of new cccDNA. The article outlines the structural organization and functional roles of the HBV core protein, emphasizing its central importance in viral genome maturation. It traces the evolution of CAM discovery—from phenylpropenamides and heteroaryldihydropyrimidines to sulfamoyl benzocarboxamides (SBAs)—and highlights the sustained interest in SBAs due to their favorable structural attributes and the availability of informative co-crystal structures. Extensive structure–activity relationship studies have yielded optimized SBA derivatives with sub-micromolar EC50 values and acceptable cytotoxicity profiles, while several advanced compounds, including NVR-3778, JNJ-632, and KR-26556, exhibit potent inhibition of HBV DNA synthesis in hepatoma models. Additional strategies, such as prodrug development and targeted modification of solvent-exposed regions, have enhanced solubility and pharmacokinetic behavior. Beyond monocyclic scaffolds, sulfamoyl bicyclic and heterocyclic carboxamides broaden the chemical landscape and demonstrate promising antiviral potential. Collectively, the advances summarized in this review position sulfamoyl-based CAMs as strong candidates for incorporation into future combination regimens aimed at achieving a functional cure for chronic hepatitis B. 

The third paper (https://doi.org/10.3390/v15122395 [17]) is also a review that analyses biotechnological strategies based on gene-editing tools and RNA interference (RNAi) as emerging therapeutic options for chronic hepatitis B virus (HBV) infection. It first outlines the global HBV burden, the role of cccDNA and integrated HBV DNA in viral persistence, and the limitations of current interferon and nucleos(t)ide analog-based therapies, which rarely achieve a functional cure. The authors summarize gene-editing approaches using zinc-finger nucleases, TALENs, and CRISPR/Cas systems, highlighting their ability to target HBV cccDNA and integrated sequences, and reduce viral replication and antigen expression in vitro and in animal models. They discuss major obstacles, including efficient and safe delivery to hepatocytes, off-target editing, cytotoxicity, and the potential infection of extrahepatic cells, which may limit complete viral eradication. The review then focuses on RNAi-based therapeutics (siRNA and antisense oligonucleotides) designed to silence HBV transcripts and reduce HBsAg and other viral markers. It summarizes key clinical candidates, their molecular targets (e.g., S and X ORFs, total HBV mRNA), dosing regimens, routes of administration, and outcomes from early-phase trials, including the depth and durability of HBsAg decline. Combination regimens with nucleos(t)ide analogs, capsid assembly modulators, and pegylated interferon are examined, showing synergistic effects but also frequent post-treatment viral rebounds. The authors underline unresolved issues such as the emergence of escape mutants, incomplete functional cure rates, the limited impact on immune exhaustion, and the gap between promising animal data and human trial results. They also note that GalNAc-conjugated siRNAs and newer delivery platforms have improved liver targeting and safety profiles yet still require repeated parenteral dosing. Overall, the review concludes that gene-editing tools hold long-term potential for sterilizing cures but remain preclinical, whereas RNAi-based drugs are closer to clinical use for functional cures but are not yet optimal. The authors emphasize that, despite these advances, HBV vaccination and improved global coverage remain essential pillars of HBV elimination efforts.

The fourth paper in this Special Issue (https://doi.org/10.3390/v16020202 [18]) is an interesting review that summarizes current advances in antiviral drug development against Japanese encephalitis virus (JEV), a neurotropic flavivirus that causes severe encephalitis in Asia and the Western Pacific and still lacks specific treatment despite effective vaccines. It first outlines JEV’s epidemiology, genotypes, transmission cycles, and recent geographic expansion, emphasizing the substantial disease burden and unmet clinical need. The authors then describe JEV’s molecular biology and life cycle, detailing the roles of structural proteins (C, prM, and E) and non-structural proteins (NS1–NS5) and highlighting NS3 and NS5 as key enzymatic targets. A hypothetical target product profile is proposed, prioritizing blood–brain barrier penetration, safety in pregnancy, and low-cost, preferably oral, regimens suitable for acute infection in endemic regions. Antiviral efforts are organized into entry inhibitors, RdRp inhibitors, protease inhibitors, bioactive natural products, and host-directed agents. Entry inhibitors include peptides (P1), small molecules such as berbamine and BP34610, proteasome inhibitors, and curcumin-derived carbon quantum dots that block E-protein-mediated entry and protect mice in lethal challenge models. Nucleoside analogs (2′-C-methylcytidine, DFMA) and virtual screening-derived natural compounds are presented as RdRp inhibitors with sub-micromolar EC50 values in JEV replicon systems. NS2B–NS3 protease-targeting molecules such as CW-33, andrographolide, abscisic acid, and aloe-emodin show in vitro inhibition, though pharmacokinetics and toxicity profiles remain to be optimized. Several natural products (ouabain, enanderinanin J, baicalein, and rosmarinic acid) display potent anti-JEV activity and, in some cases, improved survival in infected mice, while the authors note development challenges related to sourcing, standardization, and formulation. Host-directed strategies include ER α-glucosidase inhibition (NN-DNJ), calcium channel blockers (manidipine and related dihydropyridines), TNF-α blockade (etanercept), minocycline, JNK inhibition (SP600125), HDAC6 inhibition (tubacin), and the potential modulation of TLR4 signaling, many of which reduce neuroinflammation and mortality in animal models. Despite this broad pipeline, most candidates remain at the preclinical stage, and few have progressed toward clinical evaluation. The review concludes that combining structure-based design, drug repurposing, high-throughput screening, and improved in vivo models will be essential to translate these findings into effective therapies for Japanese encephalitis.

The other six papers in this Special Issue are research papers. The fifth paper (https://doi.org/10.3390/v16030347 [19]) evaluates whether a second treatment cycle with GalNAc-conjugated HBV-siRNA can deepen antigen reduction and prevent rebound in an AAV-HBV mouse model. Two baseline antigenemia settings were established (≈10^3^ and 10^5^ IU/mL HBsAg), and animals received one or two 12-week treatment cycles separated by an 8-week washout. A single cycle induced a rapid, biphasic HBsAg decline followed by rebound, independent of baseline levels. Retreatment achieved significantly greater suppression: in mid-titer mice, HBsAg and HBeAg became undetectable and remained suppressed, while high-titer mice showed an additional ≈1-log decline without rebound. Liver IHC confirmed deeper reductions in intrahepatic HBsAg and HBcAg after two cycles. ALT elevations during retreatment indicated immune-mediated clearance rather than hepatocyte turnover. Bulk RNA-seq revealed marked transcriptional activation only after retreatment, especially in mid-titer mice (425 DEGs), with the upregulation of Gpnmb, Mmp12, and Trem2, suggesting macrophage activation and tissue repair programs. Flow cytometry showed increased PD-1 and TIGIT expression on CD4 T-cells and higher frequencies of CD4 Tregs after retreatment, consistent with antigen-driven T-cell activation and exhaustion dynamics. Collectively, the data demonstrate that a second HBV-siRNA cycle yields deeper, more durable antigen suppression and enhances intrahepatic immune responses, supporting staged or combination RNA-based therapeutic strategies.

The sixth paper in this Special Issue (https://doi.org/10.3390/v16040522 [20]) is a prospective single-center study that investigates the prevalence of peripheral neuropathy (PN) in chronic hepatitis C (HCV) infection without mixed cryoglobulinemia and evaluates its reversibility following direct-acting antiviral (DAA) therapy. Forty HCV-infected individuals, including nine with HIV co-infection, and twelve healthy controls underwent electroneurography (ENG) and intraepidermal nerve fiber density assessment by skin biopsy at baseline, with repeat testing one year after sustained virological response (SVR). At baseline, PN was detected in 22.5% of HCV-mono-infected patients and 44% of HCV/HIV-co-infected patients, with polyneuropathy or mononeuropathies documented by ENG. Intraepidermal nerve fiber density was significantly lower in all HCV-infected groups compared with controls (*p* = 0.0067), indicating subclinical small-fiber damage. One year after SVR, nerve fiber density improved in both mono- and co-infected groups, reaching values comparable to controls, while ENG abnormalities normalized in three of seven HCV-mono-infected patients but in none of the co-infected individuals. Improvement was particularly pronounced in patients with motor–sensory polyneuropathy, who showed significant increases in nerve density (*p* = 0.0273). Univariable and multivariable analyses confirmed that HCV infection independently correlated with reduced nerve density, whereas female sex and genotype 1 were associated with higher baseline fiber counts. Overall, the findings demonstrate that HCV infection contributes to both large- and small-fiber neuropathy even in the absence of cryoglobulinemia, and that viral eradication with DAAs leads to measurable histological and physiological improvement.

The seventh paper (https://doi.org/10.3390/v16050682 [21]) comprises a multicenter propensity score analysis from the Italian PITER cohort, which evaluated whether sustained virological response (SVR) after direct-acting antiviral (DAA) therapy reduces hepatocellular carcinoma (HCC) incidence in patients with HCV-related cirrhosis. Among 1111 DAA-treated SVR patients and 307 untreated controls, the weighted 36-month HCC incidence rates were 7.0% and 10.0%, respectively, with lower cumulative rates at 12 and 24 months also favoring the SVR group. Weighted HCC incidence was 0.20% per year in SVR patients versus 0.34% in untreated individuals. In competing risk analysis, untreated participants had a 64% higher risk of HCC (SubHR 1.64), while independent predictors of HCC included male sex, older age, current alcohol use, genotype 3, platelet count ≤ 120,000/µL, and albumin ≤ 3.5 g/dL. The findings confirm that viral eradication substantially reduces, but does not eliminate, HCC risk in cirrhosis, underscoring the need for continued surveillance even after SVR.

The eighth article is a medicinal chemistry research article (https://doi.org/10.3390/v16081238 [12]) that presents the structure-guided design, synthesis, and antiviral characterization of new pyrazino [1,2-a]indole-1,3(2*H*,4*H*)-dione derivatives aimed at inhibiting RNA-dependent RNA polymerase (RdRp) activity in Flaviviridae viruses. Building on previous metal-chelating indole–diketopiperazine scaffolds, the authors systematically modified the imidic nitrogen and indole core to define structural determinants of activity. In HCV genotype 1b replicon assays, retaining the *N*-hydroxyimide motif was essential for potency, while replacement with carboxylic or acetohydroxamic groups markedly impaired antiviral activity. Introducing a 4-methyl substituent on the diketopiperazine ring improved activity in most analogs, and targeted modifications at the indole ring identified compound **36**, a 6-nitro derivative, as the lead inhibitor (EC_50_ 1.61 µM; CC_50_ 175.4 µM; SI ≈109). Compound **36** also showed activity against HCV genotypes 1a and 1b and had a high genetic barrier to resistance and only one resistance mutation was detected (NS5B T181I), consistent with its predicted RdRp binding mode. Additional derivatives demonstrated selective inhibition of dengue virus (compound **52**) and yellow fever virus (compound **78**), indicating broader anti-flaviviral potential. In silico docking and molecular dynamics supported the stable coordination of compound **36** within the RdRp active site. Overall, the study delineates key structural features that govern potency and resistance and introduces promising lead compounds and interesting structure–activity relationships for the development of pan-Flaviviridae antivirals.

The ninth paper in this Special Issue (https://doi.org/10.3390/v16081250 [22]) is an in silico study that identifies a potential covalent inhibitor for drug-resistant genotype 3 (G3) variants of the hepatitis C virus NS3/4A serine protease. The authors first analyze sequence variability in NS3/4A G3, detecting mutations at 14 ligand-binding positions, including catalytic triad substitutions H57R and S139P and several known resistance-associated substitutions (R123T, A156T, and D168Q). Homology models of 15 NS3/4A G3 variants are generated and validated structurally, then used to build a ligand-based pharmacophore from known covalent and non-covalent NS3/4A inhibitors. The pharmacophore-based virtual screening of >300 million compounds, followed by ADMET filtering and covalent docking to catalytic Ser139, yields CHEMBL569970 (cpd-217) as the top hit with favorable CovDock and MM/GBSA scores across almost all variants. Detailed interaction analysis and 100 ns molecular dynamics simulations show that cpd-217 forms a stable covalent bond to Ser139 and maintains key contacts with residues in the oxyanion hole and S1/S2 pockets, although some RAS patterns (e.g., S139P, A156T) weaken specific interactions. MM/GBSA energy decomposition reveals how resistance mutations redistribute binding energy within the pocket, explaining the reduced susceptibility of certain variants. Off-target and scaffold similarity screening (SEA, SwissTargetPrediction) indicate a novel chemotype with low predicted off-target risk. The study proposes cpd-217 as a promising covalent lead for further in vitro and in vivo evaluation against HCV G3 NS3/4A.

The tenth paper (https://doi.org/10.3390/v16121926 [23]) evaluates a library of 23 known and novel glycyrrhizic acid (GL) conjugates with amino acids and dipeptide esters as inhibitors of dengue virus type 2 (DENV-2) NS2B-NS3 protease. Using FRET-based trans-cleavage assays, several derivatives significantly reduced protease activity, and docking to the DENV-2 NS2B-NS3 active site (PDB 2FOM) identified group I compounds (**11**, **16**, **17**, **19**, and **20**) with favorable interactions at His51, Asp75, Ser135, and Gly153. Compounds **11**, **17**, and **19** showed the strongest dose-dependent inhibition, with IC50 values of 0.0134, 0.34, and 0.52 µM, respectively. In DENV-2-infected Vero E6 cells, these conjugates reduced the cytopathic effect, NS4B-positive cells, and virus yield; compound **11** was the most potent, with EC50 values of 0.034 µM for infectivity and 0.042 µM for virus yield and a selectivity index > 2000. All three compounds inhibited post-entry stages of viral replication, while compound **19** also interfered with viral entry in time-of-addition/removal assays. The work defines key NS2B-NS3 binding interactions for GL-derived scaffolds and proposes compound **11**, and to a lesser extent **17** and **19**, as promising leads for further optimization as anti-dengue protease inhibitors.

The final eleventh paper (https://doi.org/10.3390/v17010006 [24]) systematically characterizes allosteric small-molecule binding sites in inactive conformations of NS2B-NS3 proteases from eight pathogenic flaviviruses (ZIKV, WNV, YFV, JEV, and DENV-1–4). Using 92 crystallographic structures and 13,692 NS2B-NS3 sequences, the authors classify proteases into three conformational states—closed active, transient inactive, and fully opened inactive—and introduce a unified numbering scheme to compare them. Pocket prediction with ICM PocketFinder and DLID scoring identifies three non-active site pockets (AP1–AP3), located in transient and fully opened states and partially overlapping but distinct from the catalytic pocket. Structural analysis shows that AP1 and AP2 are more enclosed and less charged than the active site pocket, suggesting superior “druggability,” while AP3 appears in both closed and inactive states and offers an additional allosteric target. RMSD and B-factor analyses indicate that backbone conformations around AP1/AP2 are sufficiently conserved for structure-based inhibitor design, with DENV-2 showing the largest deviations. Large-scale sequence analysis reveals high intra-species conservation around these pockets (especially JEV AP1/AP2), but only moderate inter-species identity, implying that pan-flaviviral inhibition is challenging, whereas multiviral or virus-specific allosteric inhibitors are feasible. The work proposes AP1 and AP2 as promising target sites for future screening of allosteric inhibitors aimed at stabilizing inactive NS2B-NS3 protease states and overcoming the limitations of active site-directed compounds.

This Special Issue brings together a remarkably diverse portrait of modern antiviral research, following the path from molecular design to biological validation across hepatitis viruses and the wider Flaviviridae family. The studies presented here explore small-molecule scaffolds, covalent and allosteric inhibition strategies, RNA-targeted approaches, and the structural roots of antiviral resistance, with each one offering a different window into how viral replication can be interrupted. Several contributions use sophisticated computational tools such as pharmacophore modeling, covalent docking, and molecular dynamics to uncover hidden weaknesses in viral proteases and polymerases, while others show how effective treatment can reshape host–virus interactions, help repair virus-induced nerve damage, or reduce the long-term risk of liver cancer. Together, these papers capture a field moving quickly and creatively, where medicinal chemistry, virology, and structural biology work hand in hand to generate new ideas and tangible therapeutic leads. As these approaches evolve, they will widen our therapeutic possibilities and open new avenues for confronting both long-standing and newly emerging viral threats.
